# Supplementary material for: Capabilities and Limitations of Tissue Size Control through Passive Mechanical Forces
Source: PLoS Comput Biol. 2015 Dec 29;11(12):e1004679. doi: 10.1371/journal.pcbi.1004679 (PMC4703071; doi:10.1371/journal.pcbi.1004679)
Supplement: S1 Text — (PDF) [file pcbi.1004679.s002.pdf]

Capabilities and Limitations of Tissue Size Control Through Passive Mechanical Forces: Supporting Information – Text S1

Immunohistochemistry A *Drosophila* line expressing GAL4 under the engrailed (en) promotor and CD8::GFP under the UAS promotor was used. Immunohistochemistry on embryos was performed as described previously [1] with rabbit anti-dpERK (1:100, Cell Signaling), rat anti-DCAD2 (1:100, DSHB) and DAPI (5 µg/ml, Invitrogen DU1306), with goat anti-rat IgG 561 (1:500, Invitrogen), and goat anti-rabbit IgG 647 (1:500, Invitrogen).

Confocal microscopy Confocal z-stacks were collected using an Andor spinning disc confocal microscope with a piezo stage at 1.0 µm intervals. For each embryo, six by six grids with thirty-three percent overlap were collected for each of the four channels. Image collection was performed with MetaMorph® version 7.0.11. A costum vignetting correction algorithm was applied as a part of the stitching process.

Choice of simulation boundary condition does not significantly influence simulation results In this section we analyse the influence of the choice of boundary condition on the compartment sizes and compartment cell numbers in the simulations. We compare the results of simulations using the doubly periodic boundary conditions described in the main text to those where fixed boundary conditions are imposed on the tissue as follows. Vertices on the boundary of the tissue experience the same forces as non-boundary vertices. At each time step, after applying the forces to all vertices and updating their positions, we map the boundary vertices perpendicularly back onto the boundary. If a cell on the boundary undergoes a T2 transition, then the newly created vertex is also mapped onto the boundary. The dimensions of this fixed boundary are chosen such that the total area of the tissue equals the sum of the target areas of all cells of the tissue. The tissue occupies a total of 64 rescaled area units, or 7.744×10^3 µm^2. The length of each fixed boundary is 8 L, i.e. 88 µm.

As Fig. S1 shows, we find that imposing this alternative boundary condition gives similar predicted P compartment areas and cell numbers to those obtained using doubly periodic boundary conditions. This is true across *wt*, *en*>*dap* and *en*>*CycE* simulations.

Irregular initial conditions do not significantly influence simulation results. We next analyse the influence of the choice of initial tissue geometry on the final P compartment sizes and cell numbers in our model. In particular, we compare the results of simulations initiated with randomly generated cell shapes to those of simulations with regular hexagonal cell shapes. The random initial conditions are created as follows. First, 64 seeds are randomly placed in a spatial domain occupying 64 rescaled area units (7.744×10^3 µm^2). Second, the random seeds are mirrored along each tissue boundary to ensure periodicity, and the Voronoi tessellation of all seeds is computed. Third, we relax the Voronoi tessellation using five steps of Lloyd's relaxation algorithm [2]. Cells are then assigned to the P compartment if their centroid falls within a central vertical band of width 2.9 rescaled length units (32 µm).

Fig. S3 shows that these irregular initial conditions result in similar mean P compartment sizes and cell numbers to those obtained using our regular hexagonal initial conditions, while increasing the standard deviation in each quantity. Thus, irregularity in the initial geometric configuration does not significantly affect simulation outcomes.

Changes in initial target areas do not strongly affect simulation results. For both the boundary conditions considered in this study, the initial area of each cell A^s takes its target value A_0^s. To test whether our results depend strongly on this assumption, we ran control simulations in which the initial area of each cell was half its target value at the beginning of the simulations. We found that resulting P compartment sizes and cell numbers were not strongly affected by this modification, and compartment size control was preserved. The results of this analysis are shown in Fig. S4, and they differ from Fig. 3B in two ways. First, the tissue areas at the end of simulations vary slightly from the experimental observations made by Parker; in particular, the P compartment for the *en>cycE* perturbation is larger than for the *en>dap* perturbation. This is to be expected, since in this set of simulations most cell areas are far from their target values, hence the area energy term dominates and mitosis-induced shrinkage does not occur. Second, *en>CycE* P compartment cell numbers are smaller than observed by Parker and in the results of Fig. 3B (52 vs 60

and 72 cells, respectively), illustrating that more cells die in the simulations where cell target areas have been doubled. In the simulations summarized in Fig. S4, the overall tissue target area is a lot larger than the tissue area, and therefore more cells are removed by apoptosis (T2 swaps) to decrease the corresponding energy term. Although we consider the initial condition for the simulations with doubled initial target areas A_0^s unrealistic, with cell areas being far from their target values, the model continues to exhibit P compartment size control.

References

1. Zartman JJ, Kanodia JS, Cheung LS, Shvartsman SY. Feedback control of the EGFR signaling gradient: superposition of domain-splitting events in *Drosophila* oogenesis. Development. 2009;136(17):2903–2911.

2. Lloyd S. Least squares quantization in PCM. IEEE Trans Inform Theory. 1982;28(2):129–137.
