# Supplementary figures and images for: Capabilities and Limitations of Tissue Size Control through Passive Mechanical Forces
Source: PLoS Comput Biol. 2015 Dec 29;11(12):e1004679. doi: 10.1371/journal.pcbi.1004679 (PMC4703071; doi:10.1371/journal.pcbi.1004679)

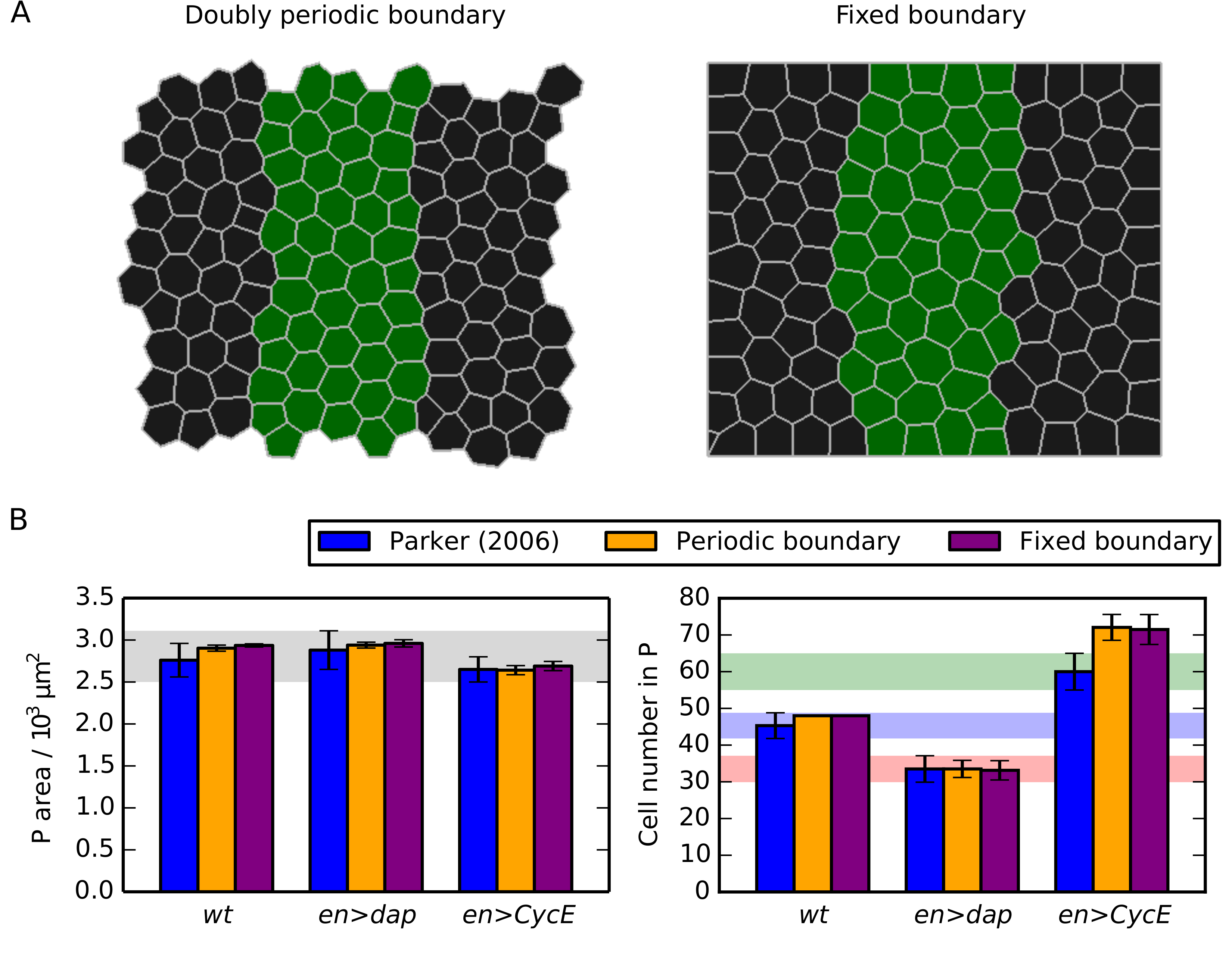

Supplement: S1 Fig — (A) Snapshots of a wt simulation at the final time point, once all cell divisions have occurred, where doubly periodic (left) or fixed (right) boundary conditions are imposed. Parameter values are listed in Table 1. (B) Comparison of P compartment areas and cell numbers for wt, en>dap and en>CycE simulations where doubly periodic or fixed boundary conditions are imposed. Mean values from 100 simulations are shown and error bars are standard deviations. Shaded areas mark the ranges of experimentally observed values and are added for reference and comparison with Figs 3, 4 and S2. (TIFF) [file pcbi.1004679.s003.tiff]

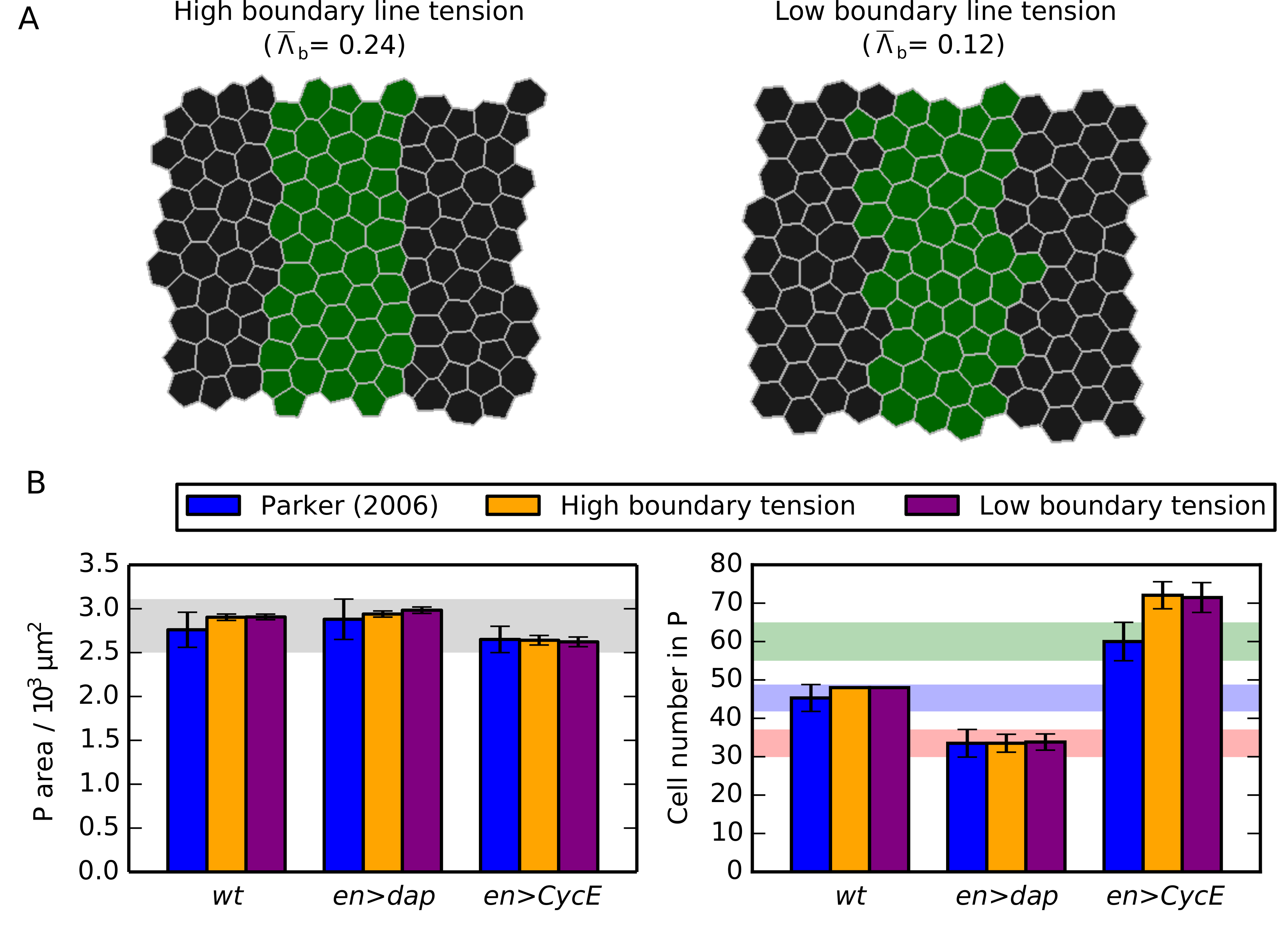

Supplement: S2 Fig — (A) Snapshots of a wt simulation at the final time point, once all cell divisions have occurred, where either a high (left) or low (right) line tension, Λ¯, is imposed at the boundary between A and P compartments. Parameter values are listed in Table 1. Compartment boundary line tension promotes cell sorting and straightness of the boundary, but does not affect compartment sizes. (B) Comparison of P compartment areas and cell numbers for wt, en>dap and en>CycE simulations where a high (left) or low (right) compartment boundary line tension is imposed. Values for Λ¯ at the compartment boundary are those given in (A). Mean values from 100 simulations are shown and error bars are standard deviations. Shaded areas mark the ranges of experimentally observed values and are added for reference and comparison with Figs 3, 4 and S1. (TIFF) [file pcbi.1004679.s004.tiff]

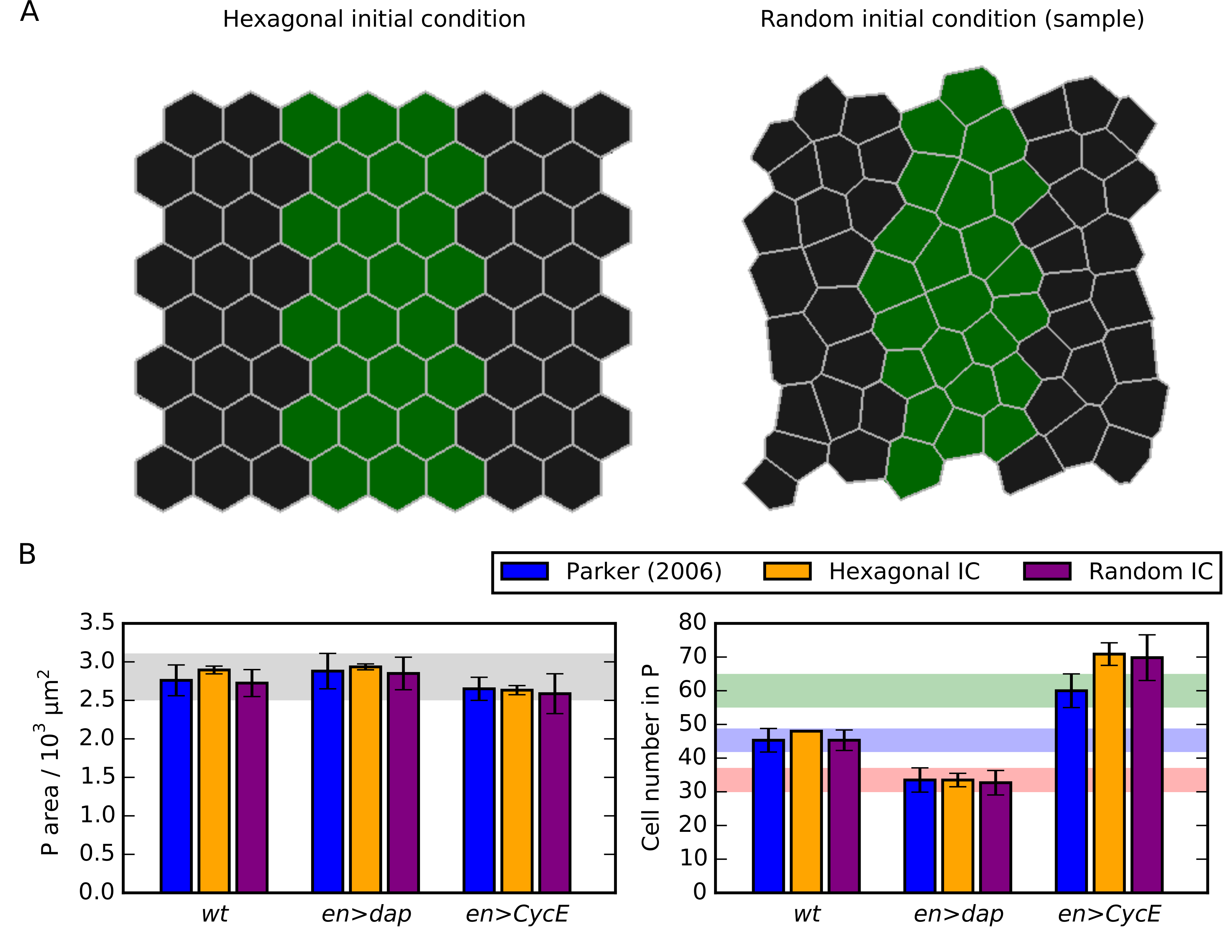

Supplement: S3 Fig — (A) Snapshots of a hexagonal (left) initial condition, and a sample random (right) initial condition, as described in S1 Text. The cells assigned to the posterior compartment occupy a similar area in both images. (B) Comparison of P compartment areas and cell numbers for wt, en>dap and en>CycE simulations where either a hexagonal or random initial condition (IC) was used. Mean values from 100 simulations are shown and error bars are standard deviations. Shaded areas mark the ranges of experimentally observed values and are added for reference and comparison with Figs 3, 4 and S1. Parameter values are listed in Table 1. (TIFF) [file pcbi.1004679.s005.tiff]

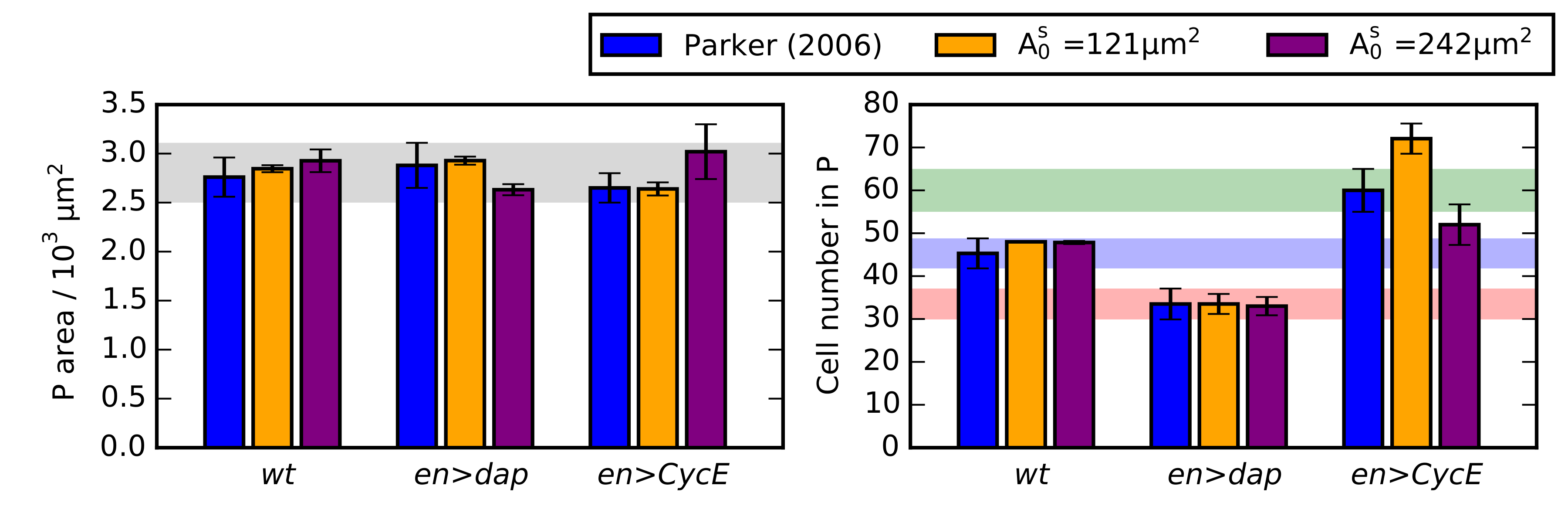

Supplement: S4 Fig — (B) Comparison of P compartment areas and cell numbers for wt, en>dap and en>CycE simulations where either initial target areas A0s=121 μm2 or A0s=242 μm2 were used. Mean values from 100 simulations are shown and error bars are standard deviations. Shaded areas mark the ranges of experimentally observed values and are added for reference and comparison with Figs 3, 4 and S1. Parameter values are listed in Table 1. (TIFF) [file pcbi.1004679.s006.tiff]
