# Supplementary material for: Capabilities and Limitations of Tissue Size Control through Passive Mechanical Forces
Source: PLoS Comput Biol. 2015 Dec 29;11(12):e1004679. doi: 10.1371/journal.pcbi.1004679 (PMC4703071; doi:10.1371/journal.pcbi.1004679)
Supplement: S1 Table — For each case of the passive mechanical model considered in Fig 3, we present the mean number of T1 swaps across 100 simulations. The indicated errors are standard deviations. (PDF) [file pcbi.1004679.s007.pdf]

wt	*en>dap*	*en>CycE*
0	0.02 ± 0.14	3 ± 1.7
